# Winter temperatures decrease swimming performance and limit distributions of tropical damselfishes

**DOI:** 10.1093/conphys/cov039

**Published:** 2015-09-18

**Authors:** Jacob L Johansen, John F Steffensen, Geoffrey P Jones

**Affiliations:** af1 Whitney Laboratory for Marine Bioscience, University of Florida, St Augustine, FL 32080, USA; af2 ARC Centre of Excellence for Coral Reef Studies, and College of Marine and Environmental Sciences, James Cook University, Townsville, QLD 4811, Australia; af3 Marine Biological Section, University of Copenhagen, Strandpromenaden 5, DK-3000 Helsingør, Denmark

**Keywords:** Abundance, distribution, metabolism, thermal window, tropical teleosts, temperature

## Abstract

Coral reefs within 10° of the equator generally experience ≤3°C seasonal variation in water temperature. Ectotherms that have evolved in these conditions are therefore expected to exhibit narrow thermal optima and be very sensitive to the greater thermal variability (>6°C) experienced at higher latitudes (≥10°N/S). The impact of increased thermal variability on the fitness and distribution of thermally sensitive reef ectotherms is currently unknown. Here, we examine site-attached planktivorous coral reef damselfishes that rely on their physiological capacity to swim and forage in the water column year round. We focus on 10 species spanning four evolutionarily distinct genera from a region of the Great Barrier Reef that experiences ≥6°C difference between seasons. Four ecologically important indicators showed reduced performance during the winter low (23°C) compared with the summer peak (29°C), with effect sizes varying among species and genera, as follows: (i) the energy available for activity (aerobic scope) was reduced by 35–45% in five species and three genera; (ii) the energetically most efficient swimming speed was reduced by 17% across all species; and (iii) the maximal critical swimming speed and (iv) the gait transition speed (the swimming mode predominantly used for foraging) were reduced by 16–42% in six species spanning all four genera. Comparisons with field surveys within and across latitudes showed that species-specific distributions were strongly correlated with these performance indicators. Species occupy habitats where they can swim faster than prevailing habitat currents year round, and >95% of individuals were observed only in habitats where the gait transition speed can be maintained at or above habitat currents. Thermal fluctuation at higher latitudes appears to reduce performance as well as the possible distribution of species and genera within and among coral reef habitats. Ultimately, thermal variability across latitudes may progressively cause sublethal changes to species performance and lead to a contraction of biogeographical range.

## Teaser

Planktivorous coral reef fishes occupy sheltered to exposed reef habitats based on their ability to swim and foraging within the current. However, climate driven fluctuations in temperature can reduce this ability to swim and direct the distribution of species to more sheltered habitats.

## Introduction

The physical and physiological capacity to maintain performance across seasonal temperatures is thought to be essential for the survival of most ectothermic species (e.g. Pörtner and Farrell, 2008; Tewksbury *et al.*, 2008; Pörtner *et al.*, 2010). However, organisms living in thermally stable environments, such as tropical coral reefs, can maximize growth, reproduction and functional performance by specializing within a narrow range of temperatures (Huey and Slatkin, 1976; Huey and Hertz, 1984; Wood and McDonald, 1997; Clarke, 2003; Pankhurst and Porter, 2003; Pörtner *et al.*, 2005, 2010). For most coral reefs, particularly at latitudes <10°N/S where seasonal temperatures vary ≤3°C (Lough, 2012), resident ectothermic organisms are predicted to be temperature specialists without the physiological capacity to cope with large temperature fluctuations (e.g. Pörtner and Farrell, 2008; Tewksbury *et al.*, 2008; Pörtner *et al.*, 2010). Several recent studies have confirmed that tropical organisms, such as coral and reef fish, are highly sensitive to minor thermal fluctuations, with temperatures merely 2–4°C above the present-day summer average causing severe reductions in performance and fitness and even death of some species (Hoegh-Guldberg, 1999; Deutsch *et al.*, 2008; Martin and Huey, 2008; Tewksbury *et al.*, 2008; Munday *et al.*, 2009; Dillon *et al.*, 2010; Johansen and Jones, 2011). As a result, thermal changes and fluctuations greater than the 3°C seasonal norm are thought to present a serious threat to the ecology, health and survival of numerous tropical coral reef species.

Some coral reef systems are naturally exposed to thermal fluctuations that are substantially greater than the global average for coral reefs (e.g. Al-Rashidi *et al.*, 2008; Donner, 2011; Lough, 2012). At latitudes >10°N/S, reefs can experience seasonal temperature fluctuations of 6°C or more (Donner, 2011; Lough, 2012). Yet, many reef organisms, such as reef fishes, that exist in both equatorial and higher latitude reefs are known to exhibit strong thermal specialization owing to their ectothermic physiology and often narrow thermal windows (Munday *et al.*, 2009; Johansen and Jones, 2011). Such thermal specialization and associated sensitivity may cause significant seasonal changes in performance at higher latitudes, shape relative patterns of fitness and reduce species survival and distribution. Thermal specialization and sensitivity would also provide a reasonable explanation for the reduced breadth of habitat conditions occupied by equatorial species at higher latitudes (following the ‘seasonal variability hypothesis’; Gaston and Chown, 1999; Addo-Bediako *et al.*, 2000). The impact of large seasonal fluctuations in temperatures on physiological performance has never been examined for tropical reef fishes, and the potential ecological implications are not well understood.

Two critical and interrelated aspects of performance in coral reef fishes are metabolic performance and swimming ability, which in combination may have major repercussions for species ecology, including settlement, distribution, foraging and predator evasion (Fields, 2001; Fisher and Bellwood, 2003; Blake, 2004; Pörtner *et al.*, 2005, 2010; Domenici *et al.*, 2008). The energy available for activity is reflected by ‘aerobic scope’, which is the range between the minimal and maximal aerobic metabolic rate that can then be directed towards critical behavioural tasks (Clarke, 2003; Korsmeyer and Dewar, 2005; Pörtner, 2009). At optimal temperature, aerobic scope affords maximal oxygen delivery to tissues and allows more strenuous activities, such as swimming (Lee *et al.*, 2003; Wells, 2005). At temperatures outside of optimum, however, the delivery of oxygen and capacity for activities may be severely compromised (Clarke, 2003; Nilsson *et al*., 2009; Pörtner, 2009).

One abundant group of fishes that are particularly reliant on high-energy activities and strong swimming abilities are planktivores. These fishes predominantly forage on small zooplankton in the water column and require adequate swimming performance continuously to overcome and manoeuvre within ambient currents (Hamner *et al.*, 1988). If the availability of metabolic energy or swimming performance changes between seasons, then it may directly impact where these species can forage and survive within and across latitudes. Additionally, previous studies have shown significant thermal sensitivity in this group of fishes, quickly losing scope of activity and the ability to swim at temperatures only a few degrees above optimum (Johansen and Jones, 2011).

This study examines the effect of increased seasonal temperature fluctuations on the metabolism and swimming performance of a range of site-attached planktivores tropical coral reef fishes that are commonly found at latitudes both above and below 10°N/S (Randall *et al.*, 1997). We hypothesize that at >10°S latitudes where seasonal temperatures differ by ≥6°C, thermally sensitive reef fishes cannot maintain equal metabolic and swimming performance during both summer high and winter low temperatures. We also hypothesize that significant reductions in metabolic performance and/or swimming ability between seasons will reduce the breadth of habitats these species can occupy. Specifically, where performance is reduced at low winter temperatures, we predict that the lowest seasonal swimming performance of a species will be correlated closely with the maximal current velocity occupied; that is, they can only persist in a habitat in which they are able to swim and forage in the water column all year round.

## Materials and methods

### Ethics statement

All animal experimentation was conducted in accordance with the Australian Animal Care guidelines and approved before implementation by the James Cook University animal ethics committee (Ethics No. A1267). Fish collections were performed under the James Cook University collection permits (Great Barrier Reef Marine Park Authority G06/20234.1 and Queensland Fisheries Permit 103256).

### Study species and location

This study focused on 10 species of planktivorous damselfish (Pomacentridae): *Chromis atripectoralis*, *Chromis ternatensis*, *Dascyllus aruanus*, *Dascyllus reticulatus*, *Neopomacentrus azysron*, *Neopomacentrus bankieri*, *Neopomacentrus cyanomos*, *Pomacentrus coelestis*, *Pomacentrus lepidogenys* and *Pomacentrus moluccensis*. These planktivorous species were selected because they are abundant across or below the 10°N/S latitudes (Randall *et al.*, 1997) where seasonal temperatures fluctuate by either ≤3°C or up to ≤6°C (Lough, 2012). These species also represent evolutionary lineages from two subfamilies and four different genera spread across the greater part of the Pomacentridae phylogenetic tree (Cooper *et al.*, 2008) and thereby provide the opportunity to examine thermal performance in closely related species as well as species separated by several million years of evolution. These species are site attached to particular coral reef locations spanning sheltered to highly exposed habitats and therefore have specific requirements for swimming performance when foraging in the water column (Randall *et al.*, 1997). Their metabolism and swimming performance must, therefore, match or exceed individual habitat conditions, and any seasonal reductions in performance are likely to limit which habitats they can occupy.

Fish collections and field studies of species distribution patterns were carried out at Lizard Island, Great Barrier Reef, Queensland, Australia (14°40 S, 145°28 E). Lizard Island has average water temperatures of 23.2°C (±0.4) in winter and 29.1°C (±0.4) in summer (based on monthly water temperature means ± SD from 1996 to 2012, Lizard Island weather station, www.aims.gov.au), equivalent to that found at many coral reefs >10°N/S latitude (Lough, 2012). Habitat-specific currents on these reefs have also been thoroughly described (Johansen, 2014), thereby allowing direct comparison between the thermal performance of a species and the current velocities encountered in the field.

### Experimental set-up

For laboratory assessment of metabolic rates and swimming abilities, a minimum of 10 individuals of each species (*n* = 118) were collected with clove oil and barrier nets from February to March 2008 in the same habitats where each species had greatest abundance (see description of field distribution below). Only adult fish were used in this study to avoid ontogenetic differences in performance, and all individuals of each species examined were within the same size range to facilitate direct comparisons between temperatures (two-way ANOVA, *F*_TL_ = 0.699, *P* = 0.708; *F*_weight_ = 0.674, *P* = 0.730; mean range across species, 5.1–7.2 cm total length (TL), 4.3–6.4 g). Fish were held in 40 cm × 80 cm × 40 cm tanks under a 13 h–11 h light–dark regimen (subjected to sunrise as beginning of daylight) and fed twice daily with commercial fish foods. Tanks were continuously supplied with filtered seawater (salinity 34 ± 0.7 ppt) at a temperature of 23 ± 0.5 or 29 ± 0.6°C (mean ± SD) in a flow-through system (controlled using automated aquarium heaters in the feeder sump tanks). All individuals were acclimated for 4–6 weeks (average 4.5 weeks) before trials began to ensure full metabolic acclimation (following the duration of acclimation published by Johansen and Jones, 2011). Test fish were fasted for 24 h before experimental trials to ensure a post-absorptive state that maximized the energy available for swimming (Niimi and Beamish, 1974). All trials were conducted within the 13 h of daylight regimen in order to match the diurnal activities of the study species.

### Performance measures

At each temperature, the metabolic performance (i.e. oxygen consumption) and swimming ability (mode and speed) were measured for five to nine individuals of each species swimming solitary in an 8.31 l clear Plexiglass Steffensen-type swimming respirometer with a working section of 9.0 cm × 26.0 cm × 10.0 cm (width × length × depth). The entire respirometer was placed within a temperature-regulated bath (±0.1°C) to ensure stable temperatures during measurements, which was regulated using a PR-5714 temperature controller (PR-Electronics, Denmark) and aquaria heaters. In addition, a PR-5511 temperature controller (PR-Electronics, Denmark) with a Pt-100 sensor and a cooling coil inside the swimming respirometer ensured that the temperature remained stable (±0.05°C) inside the swimming respirometer in spite of minor heat production from water friction during maximal swimming speed. Flow straighteners (3 mm diameter) ensured laminar flow in the working section of the respirometer, which was calibrated from 0 to 80 ± 0.5 cm s^−1^ using a digital Höntzsch TAD W30 flowmeter (mean ± SEM). Solid blocking effects of the fish in the working section were corrected following Bell and Terhune (1970) and were kept below 5% for all individuals. The flow tunnel was large enough to keep wall effects <1% following Webb (1993).

At the beginning of each trial, the respirometer was filled with temperature-controlled (23 or 29 ± 0.1°C, mean ± SEM), filtered and fully aerated seawater. Next, a fish was placed in the respirometer and left to acclimate for 4–8 h at a swimming speed of 8 cm s^−1^ until oxygen consumption of the test individual reached a steady-state level and the fish had settled into a continuous swimming rhythm. The trial was then started, and the oxygen consumption of the test fish was measured at increasing swimming speeds, with 1.0 body length per second (bl s^−1^) speed increments and a total of 20 min swimming at every speed (following Hammer, 1995). The maximal critical swimming speed was dependent on the swimming ability of the individual fish, with flow speed increments steadily increasing until the fish could no longer maintain position and was swept downstream with the flow onto a retaining grid for longer than 5 s. At this point, the total swimming time and maximal flow velocity were recorded, the experiment was stopped, and the fish was returned to its holding tank. During the trial, the fish was continuously monitored for location within the flume and swimming mode (i.e. gait transition from pectoral to pectoral-and-caudal propulsion), and the total swimming time and flow speed were recorded at the point of transition from strictly pectoral swimming to pectoral-and-caudal propulsion for longer than 5 s continuously. Gait transition is important because pectoral swimming is the swimming mode most commonly used by these species for routine tasks, such as foraging over prolonged periods (>20 min), while caudal propulsion is used for maximal speed over shorter periods (e.g. Hammer, 1995; Wainwright *et al.*, 2002; Fulton and Bellwood, 2005).

A 180 s flush, 300 s wait and 720 s oxygen measurement period were applied to all swimming trials, following the intermittent flow respirometry methodology of Steffensen *et al.* (1984) and Steffensen (1989). The flushing period ensured that the oxygen concentration within the respirometer never decreased below 85% of air saturation and removed any CO_2_ build-up, while the wait period ensured complete mixing of water before the measuring period was initiated. During the experiments, oxygen levels within the swimming respirometer were measured using a fibre-optic oxygen meter (Fibox 3; Presens Precision Sensing) and monitored with Oxyview v.5.31 (Presens) and LoliResp v.1.0 software. To reduce bacterial growth and respiration within the system, the respirometer was treated with a chlorox solution and thoroughly flushed in freshwater at the completion of every trial. This procedure ensured that background respiration remained below 5–10% of the oxygen consumed by each fish during swimming trials (measured at the end of each trial), which was subtracted from subsequent oxygen consumption calculations. After the fish had been returned to its holding tank, the empty respirometer was run for one additional 20 min cycle at 8 cm s^−1^, during which the rate of oxygen depletion (i.e. background respiration) was measured.

### Field distribution relative to performance

**Figure 1:**
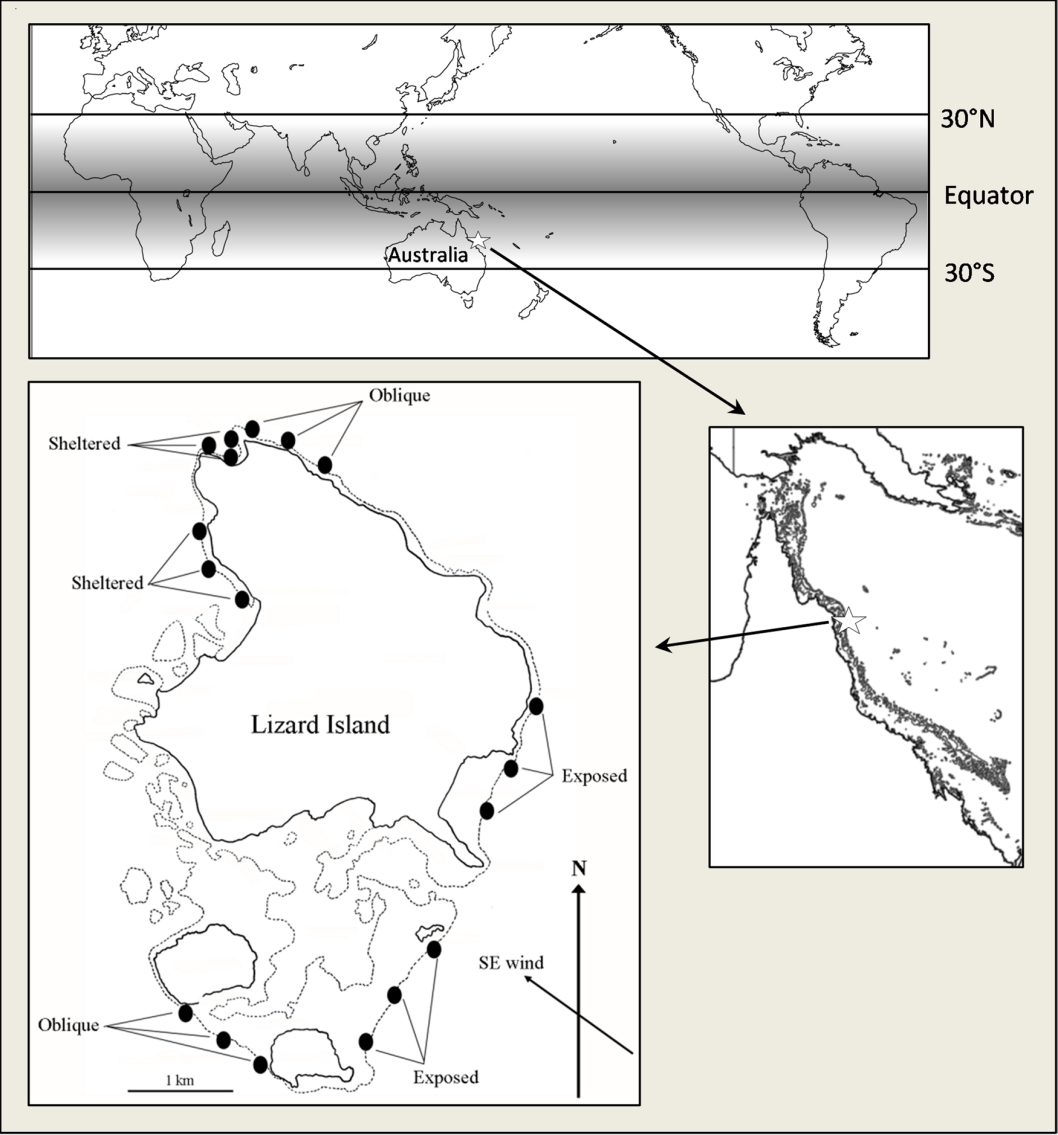
Study sites around Lizard Island, Great Barrier Reef, Queensland, Australia (14°40 S, 145°28 E). Fish abundance was examined in 54 locations across three habitat exposures, with six sites in each habitat and three depth zones in each site. Habitats were defined as exposed, oblique or sheltered relative to the prevailing south-easterly trade winds. Sites were spaced by ∼200–800 m, and depth zones were 3, 6 and 9 m depth at mid tide following the approximate depth of the crest, mid-slope and deep-slope of these habitats. Shades of grey on the global map highlight the primary latitudinal range of tropical coral reefs. The map of Lizard Island was sourced from Lizard Island Research Station. Black and grey lines indicate land and reef, respectively.

The abundance and habitat use of the 10 study species were recorded relative to ambient water currents between December and March of 2008 to 2009. Specifically, fish abundance was quantified across three habitats, with six sites at three depth zones in each site (*n* = 54 locations in total; Fig. 1). The three habitat exposures consisted of exposed, obliquely exposed and sheltered reef slope relative to the prevailing south-easterly trade winds for this location. Sites were separated by ∼200–800 m, and depth zones were 3, 6 and 9 m at mid tide. At each of the 54 locations, all adult individuals of the 10 study species were counted within a 30 m  × 3 m belt-transect.

Current velocities for these exact locations are primarily generated by the south-easterly trade winds, which are more consistent in winter (see Johansen, 2014). Ecologically, the maximal current velocity generated by each passing wave is of greater importance than average velocity, because this current imposes the greatest physical and physiological demands on resident species and may directly reduce foraging ability and dislodge sedentary organisms (Grigg, 1998; Fulton and Bellwood, 2005; Storlazzi *et al*., 2005; Madin and Connolly, 2006; Finelli *et al*., 2009). Importantly, the flow velocities imposed by continuously passing waves cannot be avoided behaviourally by resident planktivorous fishes, because this would render prolonged foraging in the water column impossible. Consequently, ecologically meaningful measures of current must include the maximal velocities that are repeatedly and continuously encountered within the spatial scales of the individual. Here, we use the maximal current velocities to calculate the currents most ecologically relevant for reef planktivores (for further detail see Johansen, 2014). We found these currents to range from 1.3 to 36.4 cm s^−1^ in deep-sheltered to shallow-exposed locations, hereafter defined as prevailing habitat currents. We used the median value of all measured maximal current velocities in combination with the 25th and 75th percentile data to demonstrate both the statistical mean and the common range of flow velocities encountered by reef organisms, as opposed to the arithmetic mean, which can be heavily skewed in either direction by episodic high velocities (i.e. outliers).

### Analyses

Performance measures provided four important indicators, as follows.

- Total cost of transport (TCOT) was used to assess the efficiency of swimming at different temperatures and speeds relative to ambient current velocities (Tucker, 1970; Schmidt-Nielsen, 1972; Fish, 1992). The TCOT is the amount of oxygen used per unit body mass per unit distance swum and is calculated for each species from the non-linear relationships between oxygen consumption and swimming speed. Specifically, TCOT (mg O_2_ kg^−1^ km^−1^) = MO_2_/(0.036 × *U* ×  *L*_T_) for each swimming speed, where MO_2_ is the oxygen consumption in milligrams of O_2_ per kilogram per hour, *U* is the swimming speed in body lengths per second, and *L*_T_ is the TL of the fish in centimetres. The relationship between TCOT and swimming speed is typically U-shaped, with the swimming speed at which TCOT is at a minimum (mTCOT) defined as the optimal speed (*U*_opt_).
- Maximal ‘active metabolic rate’ (AMR) was determined at the maximal swimming speed, while the minimal ‘standard metabolic rate’ (the basal energetic cost of maintaining bodily functions, SMR) was extrapolated from the *y*-intercept of a non-linear regression (*y* = *a*e*^bx^*) of swimming speed (in body lengths per second) vs. oxygen consumption (in milligrams of O_2_ per kilogram per hour, MO_2_), following Roche *et al*. (2013). Aerobic scope (i.e. surplus energy available for critical activities) was then calculated as: *A*_SC_ (MO_2_) = AMR – SMR.
- Gait transition (*U*_p-c_) was calculated following Brett (1964) as: *U*_p-c_ = *U* + *U*_i_ × (*t*/*t*_i_), with *U* being the penultimate current velocity before the fish transitioned from strictly pectoral to pectoral-and-caudal swimming for >5 s, *U*_i_ being ach swimming speed increment (1 bl s^−1^), *t* the length of time in the final increment where gait transition occurred, and *t*_i_ the set time interval of each swimming speed increment (20 min).
- Critical swimming speed (*U*_crit_) was calculated following the same formula, but defining *U* as the penultimate current velocity before the fish fatigued (i.e. stopped swimming) and was pinned to the downstream grid for >5 s.

The effect of seasonal temperatures was examined using two-way ANOVAs; thermal effects on metabolism (*A*_SC_) and swimming performance (*U*_p-c_ and *U*_crit_) were examined across species and genera using temperature and species as the fixed factors. This was followed by Wilks planned comparison for least-squares means for specific differences within species and phylogenetic genera. Thermal effects on mTCOT and *U*_opt_ were examined using temperature and measure as fixed factors, followed by planned comparison for least-squares means for specific differences in mTCOT and *U*_opt_ between temperatures. False detection rate was used to correct for Type I errors, following Benjamini and Hochberg (1995).

Finally, performance measures were compared with prevailing habitat current velocities. Winter current velocities reached ≤36.4 cm s^−1^ in the exposed habitat at 3 m depth, ≤21.2 cm s^−1^ in the oblique habitat at 3 m depth and ≤13.3 cm s^−1^ in all other habitats, while summer current velocities reached ≤24.8, ≤12.2 and ≤9.2 cm s^−1^, respectively (see Johansen, 2014). As all examined species had swimming performance greater than 13.5 cm s^−1^ (even during winter conditions), data were pooled across all low-current habitats to increase the strength of our statistical analysis. The 23 and 29°C swimming performance (*U*_p-c_, *U*_crit_ and *U*_opt_) of low, medium and high exposure species were compared with prevailing habitat currents during summer and winter using a two-way ANOVA with performance measure and species/habitat grouping as fixed factors, followed by a planned comparison for least-squares means for specific differences between each performance measure and habitat current velocity (corrected using false detection rate). Additionally, the minimal cost of transport (mTCOT) was compared with the actual cost (aTCOT) of swimming against habitat currents using a two-way ANOVA with temperature and measure as fixed factors, followed by a *post hoc* Tukey's HSD test for individual differences.

All data analyses and graphing were conducted using Statistica v.12.0 and SigmaPlot v.12.0, and data were square-root or log transformed where appropriate to meet assumptions for ANOVA. For all results, main effects were interpreted only when interaction terms were non-significant (*P* > 0.05).

## Results

### Seasonal thermal effects on average performance

**Figure 2:**
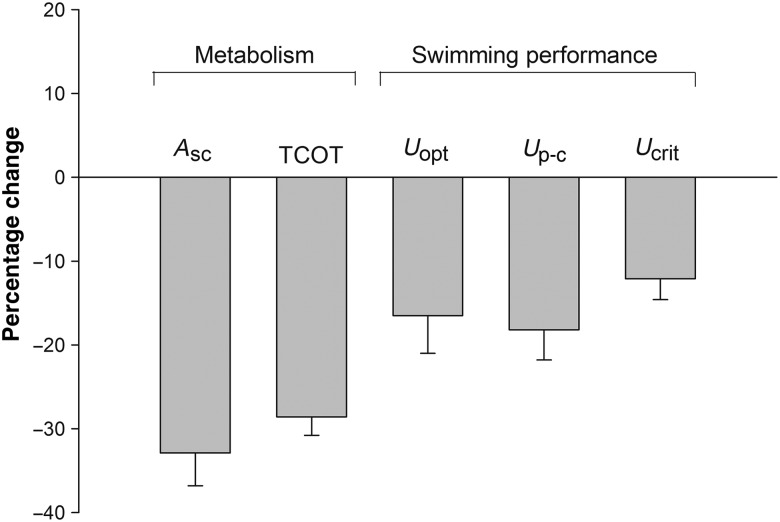
The relative change in metabolic performance and swimming ability of 10 species of coral reef damselfishes (Pomacentridae) between seasonal temperatures (29–23°C). The *x*-axis is in percent (%) and *y*-axis denotes aerobic scope (*A*_sc_), total cost of transport (TCOT), optimal swimming speed (*U*_opt_), gait transition speed (*U*_p-c_) and maximal critical swimming speed (*U*_crit_). Error bars are SEM, and all changes shown are significant (*P* < 0.01).

A change in water temperature from 29 to 23°C caused a mean 32.9 ± 3.9% (±SEM) decline in the metabolic energy available for critical activities (scope for activity, *A*_SC_) across all 10 study species (ANOVA: *A*_SC temp×species_, *F*_9,119_ = 1.81, *P* = 0.08; and main effects_temp_, *F*_1,119_ = 43.77 *P* < 0.01). This was concurrent with a mean 28.6 ± 2.2% (±SEM) reduction in minimal total cost of transport for all species combined (mTCOT_temp×measure_, *F*_1,36_ = 22.06, *P* < 0.01; and planned comparison_temp_, *t* = 4.25, *P* < 0.01; Fig. 2).

Swimming performance also declined at 23°C, with an overall reduction in the speed at which fishes transitioned from pectoral to pectoral-and-caudal swimming of 18.2 ± 3.6% (*U*_p-c temp×species_, *F*_9,119_ = 4.05, *P* < 0.01; and planned comparison_temp_, *t* = 7.08, *P* < 0.01), in optimal swimming speed of 16.5 ± 4.5% (*U*_opt temp×species_, *F*_1,36_ = 5.74, *P* = 0.02; and planned comparison_temp_, *t* = 2.40, *P* = 0.02) and in critical swimming speed of 12.1 ± 2.5% (*U*_crit temp×species_, *F*_9,119_ = 1.66, *P* = 0.11; and main effects_temp_, *F*_1,119_ = 25.77, *P* < 0.01; mean ± SEM; Fig. 2).

### Species-specific and phylogenetic effects

**Figure 3:**
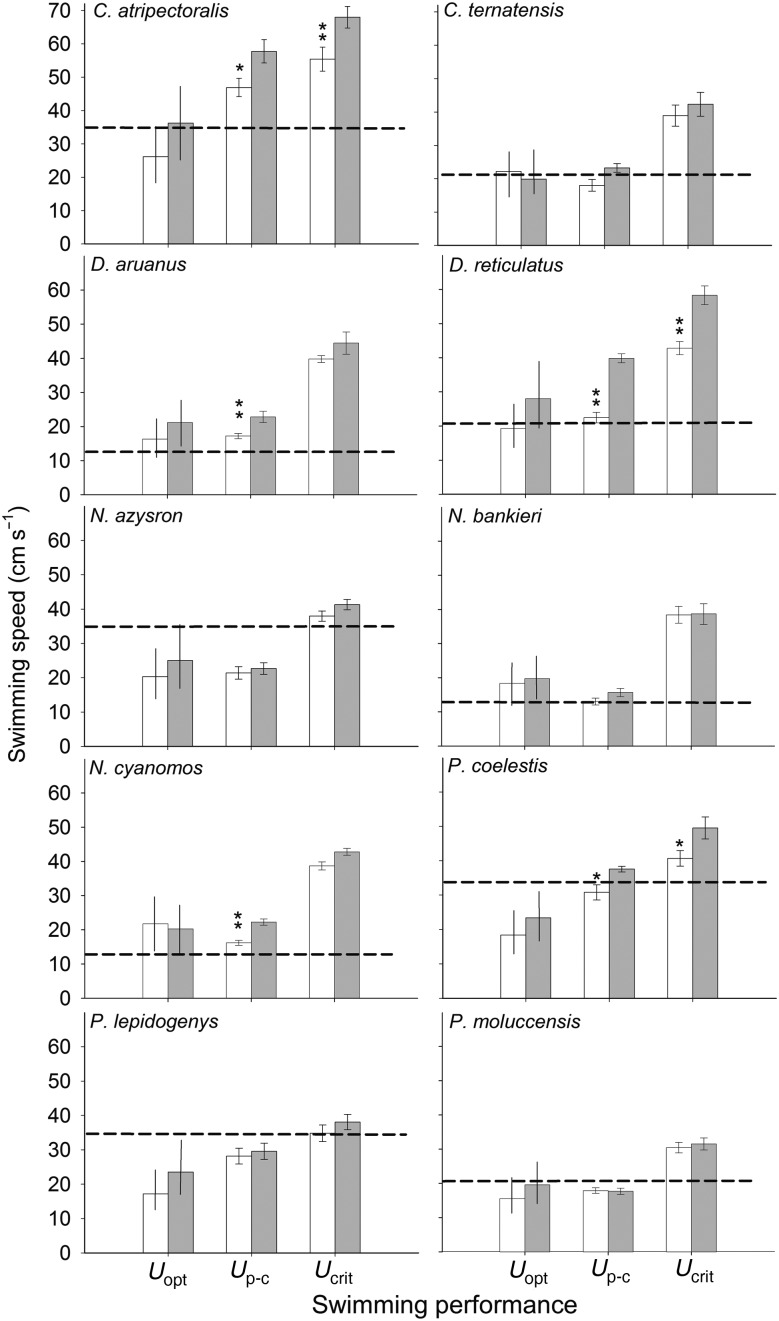
The effect of seasonal temperatures on the swimming performance of 10 species of coral reef damselfishes (Pomacentridae). For each species, the optimal swimming speed (*U*_opt_), gait transition speed (*U*_p-c_) and maximal critical swimming speed (*U*_crit_) are depicted in centimetres per second. Open and shaded bars denote performance at winter (23°C) and summer (29°C) temperatures, respectively. Horizontal dashed lines demonstrate the prevailing habitat current velocities commonly occupied by individuals in the field (recorded during winter), defined as the median of maximal wave currents. Bars around *U*_opt_ indicate the current velocities for which cost of transport remains within 5% of the lowest possible energetic cost. Bars around *U*_p-c_ and *U*_crit_ are SEM, and significant differences within species are indicated as follows: *P < 0.05 and **P < 0.01.

Overall performance was affected by temperature across all genera (Wilks planned comparison, *F*_16,319_ = 8.09, *P* < 0.01). However, the magnitude of the effect and the particular variables affected differed between species and among genera. Species of *Chromis* varied strongly in reaction to a change in temperature from 29 to 23°C. At 23°C, *C. atripectoralis* showed significant reductions in all performance measures examined, ranging from 41% in *A*_SC_, to 19% in both *U*_p-c_ and *U*_crit_, while *C. ternatensis* showed a 38% reduction in *A*_SC_ (Fig. 3; Supplemental Table S1). The two species of *Dascyllus* also varied in thermal sensitivity. *Dascyllus aruanus* showed a 26% reduction in *U*_p-c_, while reductions in the sister species *D. reticulatus* ranged from 45% in *A*_SC_ to 42% in *U*_p-c_ and 24% in *U*_crit_ (Fig. 3; Supplemental Table S1). *Neopomacentrus* was the only genus that was not significantly affected in *U*_crit_ (Supplemental Table S1). Within this genus, *N. azysron* had a 35% reduction in *A*_SC_, while the sister species *N. bankieri* showed no significant change in performance between seasonal temperature extremes. *Neopomacentrus cyanomos* was the most affected species in this genus, with a 39 and 24% reduction in *A*_SC_ and *U*_p-c_, respectively (Fig. 3; Supplemental Table S1). Finally, within *Pomacentrus*, *P. coelestis* showed a 21 and 16% reduction in *U*_p-c_ and *U*_crit_, respectively. In contrast, *P. lepidogenys* and *P. moluccensis* were the least affected, with minor but non-significant reductions in performance (Fig. 3; Supplemental Table S1).

### Distribution and abundance relative to exposure and depth

Visual surveys revealed that the distribution and abundance of all 10 study species changed gradually with habitat exposure and depth. *Neopomacentrus bankieri*, *N. cyanomos* and *D. aruanus* were most abundant in sheltered habitats and below 6 m depth in oblique and exposed habitats (>85% of all individuals) where prevailing currents remain ≤13.5 cm s^−1^ year round, and were rarely seen in habitats with higher current velocities. *Chromis ternatensis*, *D. reticulatus* and *P. moluccensis* were all common and had a high abundance at all depths in sheltered and oblique habitats, as well as below 6 m depth in exposed habitats (>85% of all individuals) with prevailing current velocities ≤21.2 cm s^−1^ year round. Only *C. atripectoralis*, *N. azysron*, *P. coelestis* and *P. lepidogenys* were commonly found within exposed habitats, with prevailing velocities consistently reaching ≤36.4 cm s^−1^ in winter (Table 1). This distinction in distribution allowed all species to be categorized as a low (≤13.5 cm s^−1^), medium (≤21.2 cm s^−1^) or high exposure species (≤36.4 cm s^−1^), which was independent of habitat location or depth.

**Table 1:** The relative abundance of 10 species of site-attached Pomacentridae relative to prevailing habitat current velocities around Lizard Island, Great Barrier Reef, Queensland, Australia

	Prevailing habitat current (cm s^−1^)			
Species abundance (%)	36.4 ± 1.6	21.2 ± 2.5	13.5 ± 2.7	Total count
*Chromis atripectoralis*	23.1^a^	55.4^a^	21.5^a^	1579
*Chromis ternatensis*	10.3	73.5^a^	16.2^a^	816
*Dascyllus aruanus*	2.1	12.6	85.3^a^	642
*Dascyllus reticulatus*	14.3	29.5^a^	56.2^a^	1018
*Neopomacentrus azysron*	37.4^a^	50.7^a^	11.9	2983
*Neopomacentrus bankieri*	0.0	0.0	100.0^a^	18
*Neopomacentrus cyanomos*	0.0	0.0	100.0^a^	74
*Pomacentrus coelestis*	52.2^a^	27.6^a^	20.2^a^	769
*Pomacentrus lepidogenys*	57.7^a^	29.5^a^	12.8	1348
*Pomacentrus moluccensis*	9.0	45.7^a^	45.3^a^	3666
Habitat exposure	High	Medium	Low	
Current range (25–75th percentile)	27–47	14–27	7–19	

Prevailing habitat currents are here defined as the median of maximal wave currents measured at 3, 6 and 9 m depth within exposed, oblique and sheltered sites relative to the south-easterly trade winds during winter (see Fig. 1) and are pooled across habitats with similar current velocities (*sensu*Johansen, 2014). ^a^ The prevailing habitat current velocities where individuals of a site-attached species are commonly found (i.e. here taken as >15% of all individuals recorded for each species). Data are based on a total of 54 transects (across three exposure regimens × six sites × three different depths), and values represent the likelihood (%) of finding a species within a specific habitat current. Note that zero values demonstrate only that no individuals were observed during transects, i.e. these species may still be present in low numbers within the habitat.

### Performance relative to habitat currents

Individuals predominantly occupied habitats where prevailing currents provided the greatest energetic efficiency for swimming; that is, species that were common in low, medium or highly exposed habitats showed no significant difference between the mTCOT and the aTCOT against habitat currents summer and winter (*F*_1,18_ = 0.07, *P* = 0.80; HSD Tukey_29°C_, *P* = 0.91; and HSD Tukey_23°C_, *P* = 0.73). Additionally, all species reached similar levels of maximal energy efficiency for swimming, because there was no significant difference in aTCOT among species occupying ≤13.5, ≤21.2 or ≤36.4 cm s^−1^ conditions (ANOVA, *F*_2,9_ = 0.18, *P* = 0.84).

**Figure 4:**
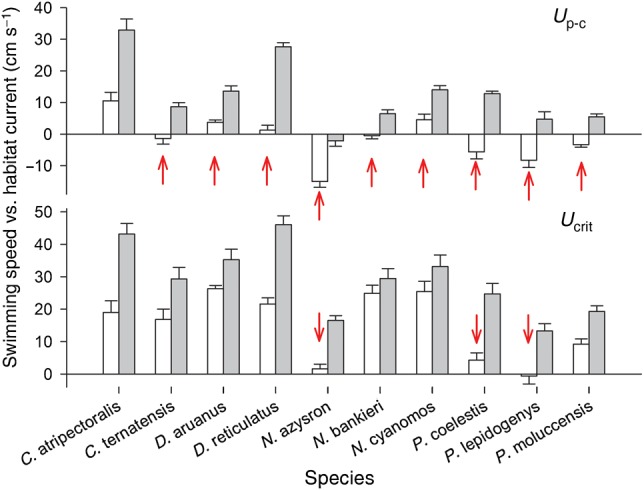
A comparison of swimming speed in 10 species of coral reef damselfishes and the prevailing current velocities commonly occupied by each species in the wild during summer and winter. Open and shaded bars denote performance at winter (23°C) and summer (29°C) temperatures, respectively. Upper graph shows the difference between gait transition speed (*U*_pc_) and prevailing habitat current velocity, defined as the median of maximal wave currents. Lower graph shows the difference between maximal critical swimming speed (*U*_crit_) and prevailing habitat current velocity. Zero values on the *y*-axis indicate that there are no differences between swimming performance and prevailing habitat current velocity. Error bars are SEM. Please note that all species are able to swim faster than the prevailing habitat current at the summer temperature. At the winter temperature, however, nine species have *U*_p-c_ and/or *U*_crit_ within 5 cm s^−1^ of prevailing habitat current velocities (indicated by arrows).

No genera or species occupied habitats with prevailing current velocities faster than they were able to swim (see Fig. 4). Furthermore, the abundance of a species within low, medium or highly exposed habitats was directly related to their lowest swimming performance recorded at winter temperatures. Specifically, at 29°C all species had *U*_pc_ and/or *U*_crit_ significantly exceeding prevailing habitat currents in their respective low, medium or high current habitats (Fig. 4 and Table 2). At 23°C, however, nine of 10 species had *U*_pc_ and/or *U*_crit_ reduced to levels directly equivalent to prevailing habitat currents (i.e. within 5 cm s^−1^; Fig. 4 and Table 2).

**Table 2:** Planned comparisons between habitat current velocities and measured swimming performance of species occupying low, medium and highly exposed habitats around Lizard Island, Great Barrier Reef, Australia

Temperature (°C)	Habitat current (cm s^−1^)	*Post hoc* planned comparison between swimming speed and habitat current velocity					
		*U*_opt_		*U*_p-c_		*U*_crit_	
		*t*_6,44_	*P*-value	*t*_6,44_	*P*-value	*t*_6,44_	*P*-value
23	13.5	1.37	0.18	1.55	0.13	6.18	<0.01
	21.2	0.97	0.34	0.96	0.34	3.57	<0.01
	36.4	4.08	<0.01	0.63	0.53	2.08	0.43
29	9.2	3.48	<0.01	4.28	<0.01	8.21	<0.01
	12.2	3.16	<0.01	3.38	<0.01	7.72	<0.01
	24.8	1.08	0.28	3.40	<0.01	6.59	<0.01

Prevailing habitat current velocities are presented as the median of maximal currents encountered during prevailing summer and winter trade winds (extrapolated from Johansen, 2014). Swimming performance is divided into the energetically optimal swimming speed (*U*_opt_), gait transition speed (*U*_p-c_) and critical swimming speed (*U*_crit_). Significant differences are accepted at *P* < 0.01 following false detection rate corrections (Benjamini and Hochberg, 1995).

## Discussion

This study is the first to show substantial differences in the performance of tropical teleost fishes between seasonal temperature extremes, which have the potential to constrain species ecology and distribution. We demonstrate that multiple coral reef damselfishes exhibit reduced metabolism and swimming performance in the cool winter waters of 23°C relative to the 29°C summer temperature commonly found at latitudes >10°S (Lough, 2012). Four evolutionarily distinct genera (*Chromis*, *Dascyllus*, *Neopomacentrus* and *Pomacentrus*), two subfamilies within this group (Chrominae and Pomacentrinae) and seven of the 10 species examined all showed significant reductions in performance. A further two species showed strong reductions in optimal swimming speed, indicating that nine of 10 species examined were sensitive to a 6°C change in seasonal temperatures. These genera naturally occur at latitudes of 22–35°N/S (Randall *et al.*, 1997), yet display reductions of up to 52% in the availability of metabolic energy and 42% in swimming performance at winter temperatures. Given that damselfishes rely on the availability of energy and the ability to swim for a majority of ecological activities (Randall *et al.*, 1997), such changes across seasonal temperature extremes were predicted to have far-reaching ecological implications. Indeed, the distribution of these sensitive planktivorous species in relationship to current velocity appeared to be linked closely to performance and energy available for swimming during the winter months, which may explain their reduced abundance in high current habitats at higher and colder latitudes (e.g. Hoey *et al.*, 2014).

The ability to perform at the cold winter temperature was not linked to phylogeny or the latitudinal distribution range of the species. Closely related sister species showed highly divergent reactions in metabolism and swimming ability to 23 and 29°C with, for example, *P. coelestis* losing 21 and 16% of *U*_p-c_ and *U*_crit_ at 23°C, while the closely related sister species *P. moluccensis* showed no significant loss of performance (see also Johansen and Jones, 2011). Likewise, the 10 species examined here differ in latitudinal range, for example from 28°N–27°S in *P. moluccensis* and *N. bankieri* to 35°N–35°S in *P. coelestis*, *N. cyanomos* and *C. ternatensis* (see the Global Biodiversity Information Facility, http://data.gbif.org/occurrences). Yet, in spite of such substantial differences in latitudinal range (i.e. incursion into colder regions), these species are also all abundant in areas where seasonal temperatures vary by ≤3°C across seasons (Randall *et al.*, 1997; Lough, 2012) and showed no clear pattern in sensitivity to the greater seasonal temperature fluctuations examined here.

The level to which a species is specialized for energetically demanding tasks, such as swimming, may be a strong predictor of sensitivity to seasonal fluctuations in temperature. The species examined here are all planktivorous and forage in the water column for prey. Specialization to fast swimming can therefore allow access to exposed high current habitats where plankton availability is greater and competition for space reduced (Hamner *et al.*, 2007), thus providing a distinct selective advantage. *Chromis atripectoralis*, *D. reticulatus* and *P. coelestis* had maximal critical swimming speeds 12–54% higher than all remaining species. However, these fast-swimming species were also the most affected by thermal changes, losing 18–24% of *U*_crit_ at 23°C compared with only 1–12% in the all of the slower-swimming species, such as *D. aruanus*, *N. bankieri* and *P. moluccensis*.

We show that reduced swimming performance during winter low temperatures may directly affect the local distribution of planktivorous coral reef fishes. Most fishes rely on swimming to conduct critical activities, and differences in swimming performance have often been used to explain species compositions across exposures (e.g. Fulton and Bellwood, 2005). Indeed, this study found a correlation between swimming speed and distribution across sheltered to exposed high current habitats. One hundred per cent of observed individuals occupy reef habitats where prevailing currents remain below their *U*_crit_ year round, and >95% of observed individuals occupy habitats where prevailing currents do not surpass their optimal swimming mode for foraging (i.e. *U*_p-c_). This pattern is strengthened by the fact that prevailing habitat currents in the locations examined are stronger during winter months (Johansen, 2014) when swimming performance is also reduced the most. A correlation between swimming performance and species distribution has major implications for species that experience substantial reductions in swimming speeds between seasonal conditions. At summer temperatures, *D. aruanus*, *D. reticulatus* and *N. cyanomos* were all able to swim and forage at velocities significantly faster than typical habitat currents encountered. At winter temperatures, however, *U*_p-c_ was reduced to velocities close to the prevailing habitat currents consistently experienced by these species, indicating that their distribution into high current habitats may be constrained by their ability to maintain swimming performance at winter lows.

Behavioural adaptations may help to alleviate some seasonal limitations in performance. A small proportion of individuals (e.g. within *P. moluccensis*) were found in highly exposed habitats where current velocities recurrently surpass *U*_crit_. Although bursts of acceleration could propel these individuals to swimming speeds well above *U*_crit_ for short periods of time (Domenici and Blake, 1997; Wu *et al.*, 2007), this is not a viable option for prolonged swimming in the water column (see Domenici and Blake, 1997; Johansen *et al.*, 2007). Rather, it is much more likely that these individuals survive in exposed high current habitats by seeking refuge closer to the substratum during unfavourable flow conditions, a behavioural strategy that has previously been shown for several other coral reef fishes (Johansen *et al.*, 2007, 2008). This behaviour would also explain why individuals of a further three species (*C. ternatensis*, *D. aruanus* and *D. reticulatus*) were occasionally observed within habitats where current velocities recurrently surpass *U*_p-c_. However, while all species may benefit from refuging near to the substratum during storms or when wave-driven currents intermittently fluctuate above sustainable swimming speeds, this strategy is also likely to impair critical ongoing activities, such as foraging, which in turn compromises their long-term fitness and ability to thrive and become dominant or abundant within more exposed habitats.

In addition to swimming performance, energetic efficiency may also drive species distributions. Most coral reefs are exposed to wave-driven currents, which are highly variable and differ greatly between sheltered and exposed habitats (see Madin *et al.*, 2006; Johansen, 2014). To forage in the water column, coral reef fishes must be able to swim continuously and be energetically efficient against these wave-driven fluctuations in current velocity. The relationship between swimming speed and energetic cost of transport is generally U-shaped, with minimal expenditure at an intermediate optimal swimming speed (*U*_opt_) and increased energetic costs of transport at both higher and lower swimming speeds. The 10 species examined here showed a relatively wide ±7 cm s^−1^ range of swimming speeds around *U*_opt_, for which the cost of transport remained within 5% of minimum. This range amply spans the fluctuating current velocities that the majority of individuals encounter in the field, thereby facilitating swimming against fluctuating waves and tidal currents with minimal excess energy expenditure. A good example was *C. atripectoralis*, for which *U*_opt_ appeared to be below habitat currents experienced at winter temperatures. Yet, this species was able to swim at speeds 32% above *U*_opt_ without increasing energetic cost of transport more than 5%, thus amply maintaining its ability to occupy these habitats without significantly increasing energetic cost of transport (see Fig. 3). Importantly, similar to other fast-swimming species, such as yellowtail kingfish (Clark and Seymour, 2006), this energetically efficient range of swimming speeds around *U*_opt_ was especially wide for species that occupy exposed high current habitats, reaching ±11 cm s^−1^ in *C. atripectoralis* vs.±6 cm s^−1^ in the low current species *N. cyanomos* (at 29°C), befitting the greater variation in flow encountered (Johansen, 2014).

Species occupying high, medium and low current habitats necessarily swim at very different speeds when foraging. Nevertheless, there appeared to be no significant difference in the energetic cost of transport (aTCOT) for the species occupying high, medium or low current habitats. To our knowledge, such energetic optimization has never previously been reported across sympatric species with different optimal swimming speeds and habitat distribution. The reason for equal energetic cost of transport for species optimized for high, medium or low current habitats is unclear but could be a result of natural limitations in energetic efficiency. Ectotherms of equal size and temperature will often have very similar basal energy requirements (Gillooly *et al.*, 2001). Specialized reef fishes of similar size may, therefore, naturally reach comparable levels of energetic efficiency and cost of swimming.

Our results indicate strong sensitivity among damselfishes to the seasonal variation in temperature commonly found on coral reefs at latitudes >10°S. Sensitivity does not appear to be related to evolutionary lineage, but may relate more closely to the swimming abilities of individual species; that is, specialization for fast, efficient swimming may confer increased thermal sensitivity and reduced acclimation capacity. An interesting question, therefore, is whether those species with greatly reduced swimming performance at winter temperatures are also restricted to more sheltered habitats in colder regions (e.g. at the borders of species biogeographical distribution and latitudes). Indeed, surveys of >270 species at high latitude tropical reefs (29°S) find these species only in sheltered low current habitats (Hoey *et al.*, 2014), and recent studies suggest that tropical vagrant fishes in temperate latitudes survive better in sheltered habitats (Feary *et al.*, 2014). These findings may also explain why widely dispersed and fast-swimming species, such as *P. coelestis*, appear less able to acclimate and thus more affected by winter temperatures than more equatorial and slower-swimming species, such as *P. moluccensis*.

While fast, energetically efficient swimming may provide evolutionary benefits in thermally stable environments, this study highlights the potential for seasonal variability to drive performance and local distribution patterns of thermally sensitive species, which may lead to restricted breadth of distribution and abundance across latitudes (similar to that predicted by the ‘seasonal variability hypothesis’; Gaston and Chown, 1999; Addo-Bediako *et al.*, 2000). Importantly, the study also emphasizes that increased seasonal variability similar to that predicted under global warming (IPCC, 2013) may cause significant sublethal changes to species distribution and ecology across latitudes.

## Supplementary material

Supplementary material is available at *Conservation Physiology* online.

## Funding

This study was funded by the Lizard Island Doctoral Research Fellowship to J.L.J., the Australian Research Council Centre of Excellence for Coral Reef Studies, James Cook University, to G.P.J. and the Danish Research Council to J.F.S.

## Supplementary Material

Supplementary Data
