# Supplementary material for: Winter temperatures decrease swimming performance and limit distributions of tropical damselfishes
Source: Conserv Physiol. 2015 Sep 18;3(1):cov039. doi: 10.1093/conphys/cov039 (PMC4778443; doi:10.1093/conphys/cov039)
Supplement: Supplementary Data [file cov039supp.zip › cov039supp.docx]

**Supplemental information:**

**Supplemental Table S1.** Post hoc planned comparisons of aerobic scope (A_SC_), gait transition speed (U_p-c_) and critical swimming speed (U_crit_) between seasonal temperatures of 23 and 29ºC. At 23ºC, 50% of species showed significant reductions in aerobic scope. Likewise, 50% of species had reductions in swimming performance. Significance was accepted at p≤0.04 following FDR corrections (Benjamini and Hochberg 1995).

**Table S2:** Post hoc planned comparisons of size differences (total length and weight) within species across temperatures. No significant difference was found for any of the species examined.

| **planned comparison of fish size at 23°C and 29°C** | | | |  |  |  |  |  |
| --- | --- | --- | --- | --- | --- | --- | --- | --- |
| species | **total length (cm)** | | | | **weight (g)** | | | |
|  | estimate | S.D. | t | p | estimate | S.D. | t | p |
| *C. atripectoralis* | -0.470286 | 0.428134 | -1.09845 | 0.274671 | -0.120416 | 0.073240 | -1.64413 | 0.103321 |
| *C. ternatensis* | -0.645667 | 0.442750 | -1.45831 | 0.147921 | -0.100832 | 0.075741 | -1.33128 | 0.186156 |
| *D. aruanus* | 0.338667 | 0.442750 | 0.76492 | 0.446141 | -0.053451 | 0.075741 | -0.70571 | 0.482024 |
| *D. reticulatus* | 0.197143 | 0.406790 | 0.48463 | 0.629009 | 0.047066 | 0.069589 | 0.67634 | 0.500401 |
| *N. azysron* | -0.050000 | 0.422146 | -0.11844 | 0.905957 | -0.005410 | 0.072216 | -0.07491 | 0.940435 |
| *N. bankieri* | -0.443333 | 0.422146 | -1.05019 | 0.296188 | -0.080754 | 0.072216 | -1.11823 | 0.266177 |
| *N. cyanomos* | -0.090000 | 0.428134 | -0.21021 | 0.833932 | -0.044754 | 0.073240 | -0.61105 | 0.542566 |
| *P. coelestis* | -0.060000 | 0.462438 | -0.12975 | 0.897030 | 0.012866 | 0.079108 | 0.16264 | 0.871133 |
| *P. lepidogenys* | 0.272857 | 0.390831 | 0.69815 | 0.486723 | 0.033154 | 0.066859 | 0.49587 | 0.621083 |
| *P. moluccensis* | 0.337143 | 0.428134 | 0.78747 | 0.432888 | 0.023273 | 0.073240 | 0.31776 | 0.751336 |
